# Ethnic differences in maternal dietary patterns are largely explained by socio-economic score and integration score: a population-based study

**DOI:** 10.3402/fnr.v57i0.21164

**Published:** 2013-07-08

**Authors:** Christine Sommer, Line Sletner, Anne K. Jenum, Kjersti Mørkrid, Lene F. Andersen, Kåre I. Birkeland, Annhild Mosdøl

**Affiliations:** 1 Department of Endocrinology, Morbid Obesity and Preventive Medicine, Oslo University Hospital, Oslo, Norway; 2 Faculty of Medicine, Institute of Clinical Medicine, University of Oslo, Oslo, Norway; 3 Department of Child and Adolescents Medicine, Akershus University Hospital, Lørenskog, Norway; 4 Norwegian Resource Centre for Women's Health, Oslo University Hospital, Oslo, Norway; 5 Department of General Practice, Faculty of Medicine, Institute of Health and Society, University of Oslo, Oslo, Norway; 6 Faculty of Health Sciences, Oslo and Akershus University College of Applied Sciences, Oslo, Norway; 7 Faculty of Medicine, Institute of Basic Medical Sciences, University of Oslo, Oslo, Norway

**Keywords:** dietary patterns, socio-economic status, integration level, ethnic minority groups, immigrants

## Abstract

**Background:**

The impact of socio-economic position and integration level on the observed ethnic differences in dietary habits has received little attention.

**Objectives:**

To identify and describe dietary patterns in a multi-ethnic population of pregnant women, to explore ethnic differences in odds ratio (OR) for belonging to a dietary pattern, when adjusted for socio-economic status and integration level and to examine whether the dietary patterns were reflected in levels of biomarkers related to obesity and hyperglycaemia.

**Design:**

This cross-sectional study was a part of the STORK Groruddalen study. In total, 757 pregnant women, of whom 59% were of a non-Western origin, completed a food frequency questionnaire in gestational week 28±2. Dietary patterns were extracted through cluster analysis using Ward's method.

**Results:**

Four robust clusters were identified where cluster 4 was considered the healthier dietary pattern and cluster 1 the least healthy. All non-European women as compared to Europeans had higher OR for belonging to the unhealthier dietary patterns 1–3 vs. cluster 4. Women from the Middle East and Africa had the highest OR, 21.5 (95% CI 10.6–43.7), of falling into cluster 1 vs. 4 as compared to Europeans. The ORs decreased substantially after adjusting for socio-economic score and integration score. A non-European ethnic origin, low socio-economic and integration scores, conduced higher OR for belonging to clusters 1, 2, and 3 as compared to cluster 4. Significant differences in fasting and 2-h glucose, fasting insulin, glycosylated haemoglobin (HbA_1c_), insulin resistance (HOMA-IR), and total cholesterol were observed across the dietary patterns. After adjusting for ethnicity, differences in fasting insulin (*p*=0.015) and HOMA-IR (*p*=0.040) across clusters remained significant, despite low power.

**Conclusion:**

The results indicate that socio-economic and integration level may explain a large proportion of the ethnic differences in dietary patterns.

Compared with the host populations in Europe, ethnic minority groups of non-European descent seem to be disproportionally affected by chronic disease such as type 2 diabetes mellitus (T2DM) (1, 2) and cardiovascular disease (3, 4). Migration is associated with major changes in both the environment and in behaviours that may affect disease risk. Reported dietary changes related to migration include increased intake of meat and high-fat foods (oil, meat, and milk) and decreased consumption of legumes and vegetables, but more extensive documentation is needed (5). The shift towards more unhealthy dietary habits is of particular concern when it affects women of childbearing age, as maternal nutritional status may modify the risk of later chronic disease in the offspring (6) and may thus have wider health consequences in future generations of immigrants (7).

Recent research points out that poor health outcomes in first-generation immigrants are associated with socio-economic deprivation, particularly in female immigrants (8). A review paper from the United Kingdom indicates that a low socio-economic position may explain a large proportion of the increased mortality and general health differences between groups according to ethnic origin (9). The definition of integration level varies across fields of expertise and across researchers (10), although language proficiency seems to be a common denominator. The impact of socio-economic position and integration level on the observed ethnic differences in dietary habits has received little attention, despite a growing recognition that socio-economic factors tend to be associated with both obesity (11–13) and T2DM (11, 14).

Analyses of dietary patterns, rather than single nutrients or foods, have been increasingly used in studies of chronic diseases (15, 16). Empirically derived healthy eating patterns have been associated with lower risk of T2DM in several cohort studies (17–19). Only recently, data are emerging on how dietary patterns are related to socio-economic status, ethnicity, and culture (20). The limited knowledge about dietary habits among immigrants and ethnic minority groups in general, and possible relationships with socio-economic factors and level of integration, may be attributed to inadequate reporting of ethnic profiles in larger studies (21). To our knowledge, ethnic differences in dietary habits during pregnancy and the impact of socio-economic and integration factors have not yet been explored. The aim of this study was to: (1) identify and describe dietary patterns in a multi-ethnic cohort of pregnant women; (2) explore ethnic differences in odds ratio (OR) for belonging to a dietary pattern, when adjusted for socio-economic status and integration level; and (3) examine whether the dietary patterns were reflected in levels of biomarkers related to obesity and hyperglycaemia.

## Experimental methods

### Subject recruitment and ethical approval

The STORK Groruddalen study is a population-based cohort study of 823 healthy pregnant women attending the Child Health Clinics (CHC) for antenatal care in three administrative city districts in the area of Groruddalen, Oslo, Norway, May 2008–May 2010 (22). The study and its methods have been described in detail elsewhere (22). Information about the study was widely distributed in the three city districts of Groruddalen prior to the start of the study. General practitioners were asked to refer pregnant women to the CHC as early as possible. Midwives and research staff recruited the women at their first visit to the CHC in early pregnancy. All information material and questionnaires were translated into eight languages (22) and quality controlled by bilingual health professionals. Professional interpreters were used when needed. Women were eligible if they: (1) lived in one of the three city districts of Groruddalen; (2) planned to give birth at one of two study hospitals; (3) were less than 20 weeks pregnant at inclusion; (4) could communicate in Norwegian or any of the eight translated languages; and (5) were able to give a written consent to participate. Women with pregestational diabetes or other diseases necessitating intensive hospital follow-up during pregnancy were excluded. The participation rate was 74% (64–83% in main ethnic groups) and 59% of the participants were of non-Western origin [for flow chart, see method paper (22)]. The sample was found representative for the population of pregnant women and the major ethnic minority groups living in the Groruddalen area who attend the CHCs (75–85% of the pregnant population) (22, 23). The study was approved by The Regional Ethics Committee and The Norwegian Data Inspectorate.

### Ethnic origin

Information about ethnic origin was collected in gestational week 12±2, on average (visit 1). Ethnicity was defined as the country of birth if ethnic Norwegian, first-generation immigrant or second-generation European immigrant, and the participant's mother's country of birth if second-generation non-European immigrant (only 6.6% (*n*=50) of the sample were second-generation immigrants) (24). Due to small numbers in some groups and to retain power in the statistical analyses, ethnic origin was merged into the region categories ‘Europe’, ‘South and East Asia’, and ‘The Middle East and Africa’. The category ‘Europe’ (*n*=352) consisted mainly of ethnic Norwegians (83%) and Eastern Europeans (11.6%). The remaining 5.4% were of Nordic, other European, or Western origin. The category ‘South and East Asia’ (*n*=231) consisted mainly of Pakistanis (52.4%), 24.7% from Sri Lanka, and 17.3% from East Asia. The remaining 5.6% were of other South Asian origin. The ‘Middle East and Africa’ category (*n*=174) consisted of women from Iraq (20.1%), Turkey (14.4%), Morocco (13.2%), Africa (43.1%), and the remaining 9.2% from Central Asia (*n*=13) and South and Central America (*n*=10).

### Indicator variables for socio-economic status and integration level

Socio-economic and integration variables were collected at visit 1. Less than 1% missing values were detected on the variables concerning socio-economic status and integration level, except in the variables: number of rooms in household (182 missing; 22%) and housing tenure (47 missing; 6%). This was due to the questionnaire structure as the study personnel sometimes forgot this question. Level of missing data was similar across ethnic groups, maternal educational level and study personnel, indicating that the missing values were random. In order to replace missing values in the final data set, maternal and household socioeconomic markers were used as both predictors and dependents during a multiple imputation, generating five imputations using linear and logistic regression models. The differences between these five complete data sets, and between actual and imputed data, were minimal. One of the five imputed data sets was chosen by simple randomisation, as running pooled analyses into the principal component analysis was considered inappropriate. Ethnic Norwegians and Nordic participants were not asked questions regarding integration. Hence, they were given top scores for all integration variables.

A principal component analysis was performed on all collected socio-economic and integration-related variables (15 variables) (22). Varimax rotation with Kaiser normalisation was used to produce indicators of socio-economic status and integration level. Four variables (house type, number of rooms in household, ownership of car, and marital status) were excluded from the final analysis because of low factor loadings (<0.4) and low correlation with other socio-economic variables. All variables were graded in the same direction from low to high socio-economic status or level of integration, respectively. Two components, socio-economic score and integration score, explaining 56% of the variance, were recognised in the material in agreement with the scree plot and Kaiser's criterion. Both components had high reliability with Cronbach's *α* >0.7. Factor scores of each participant were saved through the regression method.

The socio-economic component was mainly defined by five variables with factor loadings ranging from 0.705 to 0.584. The variables were, in decreasing order: occupation (using the International Standard Classification of Occupations (ISCO-08); educational level; tenure (defined as owning vs. renting); level of household crowding (defined as persons in household per room); and employment status (defined as not paid work vs. paid work outside home).

Variables indicating integration level were based on questions from The Oslo Immigrant Health Study (25).The integration component was mainly defined by seven variables with factor loadings ranging from 0.867 to 0.484. The variables were, in decreasing order: self-reported proficiency in the Norwegian language; need of interpreter at doctor's appointments; need of interpreter during the study interviews; duration of residence; how often visited (in any context) by an ethnic Norwegian; frequency of reading Norwegian newspapers or watching Norwegian TV; and occupation.

The individual factor scores for the socio-economic score variable ranged from −2.9 (indicating the lowest socio-economic position) to 2.6 (indicating the highest socio-economic position). The individual factor scores for the integration score variable ranged from −3.6 (indicating the lowest integration level) to 1.6 (indicating the highest integration level).

### Dietary intake

Habitual diet the previous 2 weeks was characterised using a food frequency questionnaire (FFQ) in gestational week 28±2 (visit 2). The semi-quantitative FFQ was administered by trained midwives. The FFQ was created by nutritionists for this study with combined experience in developing FFQs and knowledge of dietary habits in ethnic minority groups. The FFQ was designed to capture the frequency of intake for food items considered to modify the risk of T2DM and obesity: sugary drinks (26); rapidly absorbable carbohydrates (27) (sweet cakes, white bread, etc.); whole-grain (28); fruit and vegetables (29); beans and lentils (30); dietary fats (31) (lean vs. fatty fish and meat, fatty cakes, etc.). Frequencies of intake were given for 67 food and beverage items. The food items were fruits (1 item); vegetables (2 items); legumes (2 items); potatoes (3 items); the categories bread, cereals, pasta, rice, couscous, and other staples (6 items); yoghurt with or without added sugar (4 items); meat (4 items); fish (5 items); spreads for sandwiches (11 items); cakes, desserts, and confectionery (10 items); salty snacks (3 items); and beverages and sugar added to coffee or tea (16 items). Two questions captured between-meal snacks. Frequency of intake was captured by using either five or seven frequency intervals ranging from ‘never’ to ‘several times weekly’ or ‘never’ to ‘daily’, respectively. Portion size estimates were given for beverages and added sugar to tea or coffee only (16 items). Sugar from beverages was calculated by multiplying the volume of each beverage by mean values of sugar content (based on the Norwegian food composition table) of the specific beverage. For coffee and tea, the amount of cups per day was multiplied by number of teaspoons of sugar (6 g sugar per teaspoon) to each cup. Subsequently, all sugar content per day variables were summarised to total sugar from beverages per day.

### Dietary patterns

Six variables with a very low variance (>90% of participants gave the same response) were excluded from the cluster analysis because of their tendency to form small and special clusters. The six variables were: coffee made with a cafetière; Greek or Turkish yoghurt; low-fat yoghurt; gratinated potatoes; cereals high in sugar; and dried fruits. Variables with a low variance (80–90% of participants gave the same response) were merged with similar foods if appropriate (11 variables were merged to five new variables; for complete overview see Appendix 1). Variables on portion size for beverages were excluded. Altogether, 55 variables on frequency of intake were included in the cluster analysis (Appendix 1). Clusters were extracted by Ward's method using squared Euclidian distance, in Predictive Analytics SoftWare (PASW) version 18 (SPSS Inc., Chicago, IL, USA). The values were not standardised as the distances between values were similar in all of the included variables. The number of clusters to derive was defined by the dendrogram and by controlling for robustness of different numbers of clusters through split-half replication. Cluster solutions ranging from 2 to 8 clusters were examined. Extraction of two or four clusters was interpreted as the best solution based on the dendrogram, were recognised through split-half replication and considered robust (32). When examining intake frequency of food items in two vs. four clusters, a cluster solution of four was considered appropriate as it produced more distinct dietary patterns (32, 33).

### Biological markers and anthropometric variables

Biological markers and anthropometric variables were collected at gestational week 28±2 (visit 2). The methods for measurement of blood levels of glucose and glycosylated haemoglobin (HbA_1c_) (23), fasting insulin, and C-peptide (34) have been described in detail elsewhere. Homeostasis model assessment of insulin resistance (HOMA-IR) were estimated by the Oxford University HOMA Calculator 2.2 with fasting glucose and C-peptide concentrations (35). Total cholesterol, HDL-cholesterol, LDL-cholesterol, and triacylglycerol (TAG) was measured using slide-adapted colorimetric method, observed at 540 nm (Vitros 5.1 FS, Ortho Clinical Diagnostics) at the Department of Multidisciplinary Laboratory Medicine and Medical Biochemistry, Akershus University Hospital.

Stature was measured to the nearest 0.1 cm using a fixed stadiometer (checked against a standard meter before the start of the study and twice yearly), and body weight and percent total body fat were measured with Tanita-BC 418 MA body composition analyser (22).

### Statistical analysis

A linear stepwise (bidirectional) regression analysis was conducted to assess which food items that explained most of the variation in the clusters. Associations between intake frequencies of food items within the dietary patterns were analysed through Chi-square tests. Characteristics of the sample across dietary patterns were analysed with ANOVA if the variable was continuous and normally distributed, Kruskal-Wallis if continuous and non-parametric, and Chi-square tests if categorical. Multinomial regression analysis using main effects was performed to explore the association between ethnic origin and the dietary patterns, and adjusting for socio-economic and integration level. Fasting insulin and HOMA-IR were log-transformed to attain normally distributed data. Univariate general linear models (GLM) were executed to analyse the difference of biomarker levels within the dietary patterns while adjusting for age, percent total body fat, ethnic origin, socio-economic score, and integration score. PASW Statistics 18 (SPSS Inc., Chigago, IL, USA) was used in all statistical analyses.

## Results

Of the 823 women included in visit 1, 772 attended visit 2. Due to time limitations, 15 did not complete the FFQ, leaving 757 participants with FFQ data (92% of total sample). The baseline characteristics did not differ between those with (*n*=757) and without (*n*=66) FFQ data at visit 2 (data not shown). Fifty-nine percent belonged to an ethnic minority group, with the largest minority groups being South Asians (25.2%) and Middle Eastern (14.9%). The sample mean age was 29.3 years [standard deviation (SD)=4.92] and the mean body fat percent at gestational week 28±2 was 37.4 (SD=6.1).

### Dietary patterns

Four robust dietary clusters were detected. Table 1 presents fractions of participants within each cluster with a frequency of intake above a given cut-off. Ten food items were excluded from this table because of either a low frequency of intake overall (fried fish, fish products, sugar-reduced jam, fish spreads for bread, natural and sweetened yoghurt) or low variance and insignificant differences between the dietary patterns (boiled potatoes, fruit juice, cutlets, and other fatty meats). The linear regression analysis showed that frequency of intake of 17 food items (of the 55 included in the cluster analysis) explained 58.7% of the variance within the dietary patterns, with the item full-fat milk (*R* ^2^=0.334) as the largest contributor. The other items were, in descending explanatory order: low-fat liver pâté and ham; skimmed milk; tea; coffee; beans and lentils; low-fat processed meat; semi-skimmed milk; egg as a sandwich spread; full-fat cheese; artificially sweetened soft drinks; lean fish; salty snacks; sweet biscuits; white bread; soft drinks with sugar; and chocolate.

**Table 1:** Fractions (%, or otherwise stated) of weekly or daily frequency intake of foods and beverages within the clusters

Variables	Cluster 1 (*n*=125)	Cluster 2 (*n*=199)	Cluster 3 (*n*=183)	Cluster 4 (*n*=250)	P*
Beverages (≥5 times/week)					
Soft drinks with sugar	28.8	12.6	22.4	16.8	0.002
Soft drinks with artificial sweeteners	5.6	6.0	7.1	21.2	<0.001
Full-fat milk	69.6	5.0	6.0	0.8	<0.001
Semi-skimmed milk (1.5% fat)	13.6	55.8	33.9	17.2	<0.001
Semi-skimmed milk (0.5% fat)	4.0	8.0	10.9	24.8	<0.001
Skimmed milk (0.1% fat)	2.4	2.5	4.4	20.0	<0.001
Tea	63.2	56.3	30.6	27.6	<0.001
Coffee (filtered)	12.0	9.0	10.9	30.4	<0.001
Added sugars to tea or coffee (≥1 tsp.)	69.0	50.0	41.0	32.0	<0.001
Sugar from beverages (g) median (25, 75%)†	28.00 (15.4, 38.8)	18.20 (8.4, 28.8)	19.66 (10.0, 35.8)	18.40 (8.8, 29.8)	<0.001
Bread and cereals					
White bread (daily)	40.8	3.5	44.8	4.0	<0.001
Wholemeal bread (daily)	39.2	67.8	7.1	74.8	<0.001
Cereal, low in sugar (≥5 times/week)	8.0	10.1	4.4	10.4	0.119
Cereals, high in sugar (≥3 times/week) †	8.8	3.5	4.9	3.2	0.083
Bread spreads (≥5 times/week)					
Full-fat cheese	32.0	37.7	29.5	47.6	0.001
Low-fat cheese	9.6	7.5	3.3	11.2	0.024
Liver pâté and fatty sandwich meats	2.4	6.0	4.4	13.6	<0.001
Low-fat liver pâté and ham	1.6	2.5	6.0	19.6	<0.001
Jam	12.0	5.5	10.4	4.4	0.014
Sweet spreads (chocolate etc.)	6.4	1.5	2.7	0.8	0.007
Mayonnaise-based salads	4.8	5.0	3.3	0.8	0.048
Eggs as spread	17.6	12.6	6.6	4.4	<0.001
Fruits and vegetables					
Fruit and berries (≥ 2 times/day)	43.2	48.7	35.5	55.2	0.001
Raw vegetables (daily)	28.8	37.7	25.7	32.8	0.073
Heat-treated vegetables (daily)	20.8	25.1	14.8	22.4	0.084
Beans and lentils (≥5 times/week)	8.8	5.5	2.7	1.2	0.002
Meats and fish					
Meat filets (≥5 times/week)	11.2	8.5	12.0	7.2	0.314
Low-fat processed meat (≥3 times/week)	9.6	2.0	10.9	14.8	<0.001
Lean fish (≥3 times/week)	10.4	8.0	7.7	5.6	0.408
Fatty fish (≥3 times/week)	15.2	11.6	11.5	5.6	0.019
Cooking practices					
Fried – pan or wok (daily)	21.6	15.6	19.1	10.4	0.017
Deep-fried (≥3 times/week)	8.8	3.5	3.8	0.8	0.001
Stable foods					
Rice, pasta, regular (≥5 times/week)	21.6	12.6	26.8	7.6	<0.001
Wholemeal pasta, unpolished rice (≥3 times/week)	8.8	12.1	3.8	9.6	0.036
Chips (≥3 times/week)	8.0	3.5	7.7	0.8	0.001
Confectionery, cakes, desserts, and snacks (≥3 times/week)					
Chocolate	28.0	17.6	28.4	42.4	<0.001
Cakes	18.4	9.0	15.3	14.4	0.094
Sweet biscuits	24.8	12.1	14.2	4.8	<0.001
Sweet buns/bakery products	12.8	3.5	4.4	5.2	0.003
Waffles	4.8	2.0	1.1	0.4	0.017
Ice-cream	10.4	16.1	19.1	20.0	0.107
Dessert, pudding	9.6	1.0	1.6	1.6	<0.001
Dried fruit†	16.8	10.1	6.6	6.0	0.003
Fat-reduced snacks	5.6	1.5	3.3	0.8	0.023
Salty snacks	8.0	2.5	7.1	7.2	0.105
Nuts	25.6	16.1	9.3	10.4	<0.001

* Chi-square tests where percentages are presented, and Kruskal–Wallis test where median (25, 75 percentiles) are presented.

† Not included in the cluster analysis (presented here to clarify the differences across the dietary patterns).

Cluster 1 was characterised by frequent intake of full-fat milk, high sugar intake from beverages and frequent intake of dried fruits and nuts. The frequencies of eating fruit and vegetables were about average compared with the other dietary patterns, while these women had the most frequent intake of beans and lentils. The use of white bread and wholemeal bread in cluster 1 was equally frequent. Cluster 1 was also characterised by the most frequent intake of eggs as a sandwich spread, jam and chocolate spreads, sweet biscuits and sweet bakery products. Women in cluster 2 reported the most frequent intake of vegetables, the second most frequent intake of fruit and berries, and used mainly semi-skimmed milk. Daily use of bread was reported less frequent than that in cluster 4, but wholemeal bread was preferred. Cluster 2 was also characterised by frequent use of added sugar to tea or coffee, but a lower intake of confectionery and snacks as compared to the other three clusters. Women in cluster 3 reported the least frequent intake of fruits and vegetables and of any type of milk. Daily use of bread was also reported by the lowest proportion among women in cluster 3 and if used, white bread was preferred. Also, the frequency of using jam was high. Furthermore, the women in cluster 3 reported the most frequent intake of polished rice and pasta. The highest proportion daily eaters of bread was found in cluster 4 and the most frequent use of wholemeal bread. Subsequently, they also reported the most frequent use of spreads in general, but cheeses and meats were preferred over sweet spreads. Cluster 4 had the most frequent intake of semi-skimmed and skimmed milk, soft drinks with artificial sweeteners and coffee. Women in this cluster also reported the lowest intake of added sugars to tea and coffee, and had the most frequent intake of fruit and a relatively frequent intake of vegetables. However, these women also reported the most frequent intake of chocolate. Cluster 1 may be regarded the unhealthier dietary pattern of these four, followed by cluster 3. Cluster 4 could be considered relatively healthy overall, followed by cluster 2. However, both these dietary patterns had elements that could be considered unhealthy.

### Characteristics of the sample

Selected characteristics of the sample grouped by dietary patterns are presented in Table 2. Small, but significant, differences in age and percent total body fat were found across the dietary patterns. Among first-generation immigrants only, the duration of residence was shortest in cluster 1, intermediate in clusters 2 and 3, and longest in cluster 4. The socio-economic score and integration score followed a similar pattern with lowest scores in cluster 1, but the large variation in SDs indicates diverse individual scores. Also, an association between ethnic origin and the dietary patterns was observed. European origin was mainly associated with cluster 4 (57.1%, *n*=201) and these women were least represented in cluster 1 (6%, *n*=21). Women of other ethnic origins fell almost equally into all four clusters, although origin from South and East Asia (11.7%, *n*=27) and the Middle East and Africa (12.6%, *n*=22) was least frequently observed in cluster 4.

**Table 2:** Selected characteristics of the sample by dietary patterns

		Cluster 1 (*n*=125)		Cluster 2 (*n*=199)		Cluster 3 (*n*=183)		Cluster 4 (*n*=250)		
						
	*n*	Mean	SD	Mean	SD	Mean	SD	Mean	SD	P*
Age (years)	757	28.5	5.2	29.5	4.9	28.1	4.9	30.5	4.6	<0.001
BMI (kg/m^2^)	752	27.3	4.8	28.2	4.8	27.3	4.8	27.9	4.6	0.216
Percent total body fat	752	37.1	6.2	37.8	5.9	36.8	6.4	37.8	6.1	<0.001
Duration of residence in Norway (years) †	413	5	(2, 11)	7	(4, 13)	7	(2, 12)	11	(5, 20)	<0.001
Socio-economic status score	753	−0.44	0.92	−0.14	0.97	−0.11	0.96	0.46	0.90	<0.001
Integration-level score	753	−0.55	1.20	−0.10	0.97	−0.13	1.13	0.45	0.55	<0.001
Country of origin, grouped in regions (%)						<0.001				
Europe	352	6.0	17.3	19.6	57.1					
The Middle East and Africa	174	25.3	36.2	25.9	12.6					
South and East Asia	231	26.0	32.5	29.9	11.7					

Numbers are means and standard deviations, median (25, 75 percentiles), or percentages.

* ANOVA when mean and SD are presented, Kruskal–Wallis when median (25, 75 percentiles) are presented, and Pearson Chi-square when percentage is presented.

† Data only on respondents born outside Norway.

### Dietary patterns and ethnic origin

Table 3 shows ORs of belonging to the dietary patterns by ethnic origin adjusted for age and percent body fat (Model 1), when additionally adjusting for socio-economic score (Model 2) and when adjusting for age, percent body fat, socio-economic score, and integration score (Model 3). Non-Europeans as compared to Europeans had higher ORs for belonging to clusters 1–3 vs. cluster 4. Women originating from the Middle East and Africa had an OR of 21.5 (95% CI 10.6–43.7), while South or East Asia had an OR of 21.1 (95% CI 11.0–40.4) of falling into the unhealthier cluster 1 vs. the healthier cluster 4, as compared to Europeans. In the fully adjusted model, the OR for the Middle East decreased to 3.8 (95% CI 1.6–8.9) and for South and East Asia to 6.5 (95% CI 3.1–13.5). This implies that a low socio-economic status and low integration score explained a large proportion of the observed ethnic differences. The fully adjusted ORs for being in clusters 1 and 2 vs. cluster 4 for women from The Middle East and Africa were considerably lower than women of South and East Asian women, but still significant. The pseudo *R*2 improved by each model (Table 3). The likelihood-ratio test for each of the models was significant (*p*<0.0001 in each model).

**Table 3:** Multinomial regression analysis showing OR of belonging to the dietary patterns 1–3 compared with cluster 4 by ethnic origin (Europe as a reference)

			Cluster 1			Cluster 2			Cluster 3			Cluster 4†	
							
	*r* ^2^*	*n*	OR	95% CI		OR	95% CI		OR	95% CI		OR	*P* ‡
Model 1§	0.253												<0.001
Country of origin													
Europe†		350	1			1			1			1	
The Middle East and Africa		172	21.5	10.6	43.7	9.9	5.6	17.7	6.1	3.4	11.2	1	
South and East Asia		231	21.1	11.0	40.4	8.8	5.2	14.9	6.7	3.9	11.4	1	
Model 2§	0.269												<0.001
Country of origin													<0.001
Europe†		350	1			1			1			1	
The Middle East and Africa		172	11.8	5.	25.3	7.2	3.8	13.	4.1	2.2	7.9	1	
South and East Asia		231	16.4	8.5	31.8	7.6	4.4	13.1	5.8	3.4	10.0	1	
Model 3§	0.305												<0.001
Country of origin													<0.001
Europe†		350	1			1			1			1	
The Middle East and Africa		172	3.8	1.6	8.9	3.8	1.8	7.7	1.9	0.9	3.9	1	
South and East Asia		231	6.5	3.1	13.5	4.5	2.4	8.2	3.0	1.6	5.6	1	

* Cox and Snell.

† Reference category.

‡ Multinomial regression analysis.

§ Model 1 is adjusted for age and percent body fat, Model 2 is additionally adjusted for socio-economic score, and Model 3 is adjusted for age, percent body fat, socio-economic score and integration score.

### Biological markers reflected in the dietary patterns

The relative healthiness of the dietary patterns was to some extent reflected in the biological markers before and after adjustment for ethnic origin (Table 4). Fasting glucose, 2-h glucose, fasting insulin, HbA_1c_, and HOMA-IR were significantly different across the clusters before adjusting for ethnic origin, with the most beneficial values found in cluster 4 and no apparent trend between clusters 1, 2, and 3. However, total cholesterol varied significantly with the lowest levels found in clusters 2 and 3, while clusters 2 and 4 had significantly lower values for TAG. HDL- and LDL-cholesterol did not vary significantly across the patterns. After adjustment for ethnic origin, despite low power, fasting insulin and HOMA-IR remained significant markers, while TAG was borderline significant. When adjusting for socio-economic score and integration score instead of ethnic origin, to increase power, differences in fasting insulin, HbA_1c_, and HOMA-IR remained significantly different across the dietary patterns, while fasting glucose and TAG was borderline significant (Table 5).

**Table 4:** Biological markers across the four dietary patterns. Values are adjusted means and standard error (SE), adjusted for age and percent total body fat

		Cluster 1 (*n*=125)		Cluster 2 (*n*=199)		Cluster 3 (*n*=183)		Cluster 4 (*n*=250)			
							
	*n*	Mean	SE	Mean	SE	Mean	SE	Mean	SE	*P* *	*P* †
Fasting glucose (mmol/L)	754	4.83	0.05	4.87	0.04	4.90	0.04	4.72	0.04	0.005	0.354
2-h glucose (mmol/L)	745	6.34	0.13	6.38	0.10	6.20	0.11	5.99	0.09	0.022	0.108
Fasting insulin (pmol/L)‡	742	62.09	1.05	57.54	1.04	62.09	1.04	50.47	1.03	<0.001	0.015
HbA_1c_ (%)	742	5.27	0.03	5.19	0.02	5.19	0.02	5.11	0.02	<0.001	0.402
HOMA-IR‡	742	1.67	1.03	1.58	1.03	1.68	1.03	1.51	1.02	0.016	0.040
Total cholesterol (mmol/L)	752	6.23	0.10	6.07	0.08	6.08	0.08	6.34	0.07	0.033	0.149
HDL-cholesterol (mmol/L)	752	1.90	0.04	1.91	0.03	1.88	0.03	1.97	0.03	0.137	0.381
LDL-cholesterol (mmol/L)	741	3.51	0.09	3.37	0.07	3.38	0.08	3.56	0.07	0.158	0.390
TAG (mmol/L)	752	2.06	0.07	1.90	0.05	2.05	0.05	1.96	0.07	0.064	0.133

* Univariate GLM adjusted for age and percent total body fat (df=5).

† Univariate GLM adjusted for age, percent total body fat and ethnic origin (df=13) (data not shown).

‡ Values are converted back from transformed data.

**Table 5:** Biological markers across the four dietary patterns. Values are adjusted means and standard error (SE), adjusted for age, percent total body fat, socio-economic score and integration score

		Cluster 1 (*n*=125)		Cluster 2 (*n*=199)		Cluster 3 (*n*=183)		Cluster 4 (*n*=250)		
						
	*n*	Mean	SE	Mean	SE	Mean	SE	Mean	SE	P*
Fasting glucose (mmol/L)	754	4.79	0.05	4.87	0.04	4.89	0.04	4.75	0.04	0.076
2-h glucose (mmol/L)	745	6.28	0.14	6.37	0.10	6.19	0.11	6.04	0.10	0.164
Fasting insulin (pmol/L) †	742	60.95	1.05	57.41	1.04	60.95	1.04	51.40	1.04	0.007
HbA_1c_ (%)	742	5.25	0.03	5.19	0.02	5.18	0.02	5.13	0.02	0.014
HOMA-IR†	742	1.69	1.04	1.59	1.03	1.67	1.03	1.50	1.03	0.025
Total cholesterol (mmol/L)	752	6.36	0.11	6.10	0.08	6.10	0.08	6.24	0.08	0.132
HDL-cholesterol (mmol/L)	752	1.94	0.04	1.92	0.03	1.89	0.03	1.94	0.03	0.654
LDL-cholesterol (mmol/L)	741	3.59	0.10	3.39	0.07	3.39	0.08	3.49	0.07	0.269
TAG (mmol/L)	752	2.06	0.07	1.91	0.05	2.08	0.05	1.95	0.05	0.058

* Univariate GLM adjusted for age, percent total body fat, socio-economic and integration scores (df=7).

† Values are converted back from transformed data.

## Discussion

To our knowledge, this is the first study in Europe exploring ethnic differences in dietary patterns of pregnant women and the impact of socio-economic factors. Four major dietary patterns with a varying degree of healthiness were identified in this multi-ethnic population of pregnant women in Oslo, Norway. The described dietary patterns were strongly associated with ethnic origin, where non-Europeans had higher OR for belonging to clusters 1, 2, and 3 which were interpreted as being unhealthier. However, the OR values decreased substantially after adjusting for socio-economic and integration level scores. The importance of the nutritional value of the clusters was supported by differences in biological markers associated with a dysmetabolic state. Thus, these findings may imply that unhealthy dietary practices seen in ethnic minority groups to a certain extent may be attributable to socio-economic status and integration level rather than to ethnic factors *per se*.

The dietary patterns showed large differences in frequency of intake of food items that are good sources of dietary fibre, different types of milk, sweets and added sugars to beverages. Due to the design of the questionnaire, with only frequencies for most food items, it is difficult to interpret possible differences in dietary fat quality and total fat content. Clusters 1 and 3 showed many similarities with dietary patterns named ‘Western’ in previous studies. Similarly, clusters 2 and 4 had elements of ‘healthy’ or ‘prudent’ patterns (20, 33). However, both clusters 1 and 4 also had elements of a ‘sweet’ dietary pattern, although based on different food items. Cluster 1 had a high intake frequency of sweet biscuits especially, and relatively high intakes of cakes, sweet buns and bakery products, waffles and desserts. Cluster 4 had a high intake frequency of chocolate and relatively high intakes of cakes and ice-cream.

Furthermore, no apparent differences in intake of red meat could be seen based on these results, and the overall intake frequency of fish and vegetables was rather low. Thus, as none of the clusters could be considered analogous to food patterns already described, it was decided not to assign names to the clusters.

Both socio-economic status and integration level scores explained a large proportion of the ethnic differences in dietary habits. Several studies have shown that unfavourable dietary patterns are associated with low socio-economic status in Western populations (36–39), but it is uncertain whether the same is true for ethnic minority groups. However, some studies suggest that socio-economic factors, and to some extent level of acculturation or integration, explain an important proportion of the observed ethnic differences in ill health (8, 9, 12, 40). This study suggests that socio-economic factors and the level of integration may also be of relevance to understand ethnic differences in dietary habits. However, the fully adjusted model also points to significant cultural differences in dietary preferences.

The healthier cluster 4 had lower levels of fasting insulin and HOMA-IR after adjustment for ethnic origin. In the model adjusted for socio-economic and integration scores instead of ethnicity, women in cluster 4 had in addition significantly lower HbA_1c_ and a borderline significantly lower TAG. A review on the health benefits of high dietary fibre intakes claims that especially soluble fibre may improve glycaemia and insulin sensitivity, both in diabetic and healthy subjects (41). The women in cluster 4 had the most frequent intakes of wholemeal bread, wholemeal pasta and unpolished rice, fruit and vegetables, practices that are in concordance with a possible effect on glycaemia and insulin sensitivity. However, this could not be seen in cluster 2 despite some similarities in food choice with cluster 4. A high intake of sugar-sweetened soft drinks has also shown strong and consistent associations with increased risk of T2DM (26). Intake of sugary beverages and added sugars was highest in clusters 1 and 3, in which the highest levels of insulin and insulin resistance were observed.

The participation rate across ethnic groups in this study was high. The sample is considered to be representative for the main ethnic groups included, and should probably be applicable to other European countries with similar minority populations (22). The sample size is quite large considering the broad data acquisition. The FFQ was interview-administered by trained midwives who unravelled misunderstandings throughout the interview. Individual interpretation of the questions should therefore be less prominent. The use of dietary patterns, rather than single nutrients or food items, allows for a more holistic picture of dietary habits. Cluster analyses act as a relatively objective separator, as food items are not merged together based on pre-conceived considerations in diet–disease relationships. Instead, the dietary patterns give a summary of the variance in dietary habits among the women.

Some important limitations to this study should be noted. First, the validity of the FFQ used in this study has not been tested. The FFQ was developed by researchers with extensive experience on developing FFQs. Parts of the FFQ structure and content were similar to previously validated FFQs (42, 43), particularly the beverage items, but adjustments were made to accommodate for known dietary practices of ethnic minority groups. It is possible that the FFQ has captured more variance in some ethnic groups than in others. However, all ethnic groups are represented in all four dietary patterns, and the 17 food items that explained a large amount of the variance within the patterns must therefore capture practices that are less culturally laden. The differences in biological risk factors across the clusters also add support to the validity of the dietary patterns.

Another possible limitation to the interpretation of the findings is that the material could not distinguish any predominantly healthy or prudent dietary pattern. Derivation of a larger number of clusters could have created more homogeneity within each of the clusters, but further separation was limited by the sample size and subsequently the power to adjust for confounders. Ethnic groups were also merged into quite heterogenic categories due to power considerations. Still, the relatively low numbers lead to low precision of the ORs, as the confidence intervals became wide, and limited the possibility of adjusting for additional factors. Furthermore, the cross-sectional design does not allow for considerations of temporality. However, as risk factors were not known to these otherwise healthy, pregnant women at the time of the FFQ interview, reverse causation is not likely to be a dominant factor.

Despite some acknowledged methodological weaknesses, the study adds important knowledge regarding dietary habits in multi-ethnic populations of pregnant women, and particularly how these dietary patterns may be associated with socio-economic status, integration level, and biological risk factors. Our findings indicate that socio-economic status and integration level may influence the healthiness of dietary habits to a larger extent than ethnic origin *per se*. To offset the slow knowledge progression on ethnic differences in ill health, further research is needed on the development of valid methods for dietary assessment in ethnic minority groups. Also the impact of socio-economic status, integration level, and ethnic origin on dietary habits warrant further studies, as well as diet–disease relationships in multi-ethnic populations.

## Appendix

**Appendix 1:** Overview of all variables included in the cluster analysis List of variables included in the cluster analysis. The right column describes variables that were merged to form new variables (e.g. Artificially sweetened soft drinks) to be included in the cluster analysis

Variables included in cluster analysis	Merged variables consists of:
Cola flavoured soft drinks with sugar	
Other soft drinks with sugar	
Artificially sweetened soft drinks	Artficially sweetened cola flavoured soft drinks; Artificially sweetened other soft drinks; Artificially sweetened fruit drinks
Fruit drinks and other with sugar	
Fruit juice (without added sugar)	
Full fat milk	
Semi-skimmed milk (1.5%)	
Skimmed milk	Semi-skimmed milk (0.5%); Skimmed milk (0.1%)
Tea	
Coffee	
Sugar added to tea or coffee	Added sugar to coffee; Added sugar to tea
Natural yoghurt	
Yoghurt with fruits and berries (with added sugar)	
Fruit and berries	
Unprepared vegetables	
Heat prepared vegetables	
Potatoes, boiled or baked	
Pommes frites	
Beans, lentils	
Meat filets (low in fat)	
Low fat processed meat	
High fat processed meat	
Pizza, fast food, bought outside of home	
Lean fisk	
Fatty fisk	
Fish products (fish balls, fish cake, fish pudding)	
Fish fingers, deep-fried fish	
Fried or woked (while cooking)	
Deep-fried (while cooking)	
White bread	
Wholemeal bread	
Cereal low in sugar	
Polished rice or regular pasta	
Wholemeal pasta, unpolished rice	
Full fat cheese	
Low fat cheese	
Liver pâté and meat spreads high in fat	
Low fat liver pâté and ham	
Jam	Regular jam; Light jam
Fish spreads	
Sweet spreads	
Mayonnaise-based salad spreads	
Egg as spread	
Cakes	
Sweet biscuits	
Sweet buns/bakery products	
Waffles	
Chocolate and foreign sweet snacks	Chocolate, goodies etc.; Foreign sweet snacks
Dessert or pudding	
Ice-cream	
Light snacks	
Salty snacks	
Nuts	
Unhealthy between-meal snacks	
Healthy between-meal snacks	
